# Brucellosis in Marine Mammals: Meta‐Analysis of Prevalence, Infection Patterns, Host Specificity and Zoonotic Potential

**DOI:** 10.1002/vms3.70557

**Published:** 2025-08-11

**Authors:** Nasrin Sultana Tonu, Anowar Hossain, Sadequl Islam, Bristi Kona Debnath

**Affiliations:** ^1^ Upazila Livestock Office and Veterinary Hospital, Barura Cumilla Bangladesh; ^2^ Medical Center, Hajee Mohammad Danesh Science and Technology University Dinajpur Bangladesh; ^3^ Department of Anatomy and Histology, Faculty of Veterinary and Animal Science Hajee Mohammad Danesh Science and Technology University Dinajpur Bangladesh

**Keywords:** *Brucella* prevalence, marine brucellosis, marine mammal health, molecular diagnostics, zoonotic disease

## Abstract

**Background:**

Marine brucellosis, primarily caused by *Brucella* species, presents significant ecological and public health challenges, impacting marine biodiversity and posing zoonotic risks. Despite ongoing research, data on the disease's prevalence, host specificity and transmission within marine ecosystems remains limited, underscoring the need for comprehensive analysis.

**Objective:**

This review assesses *Brucella* infection patterns in marine mammals, focusing on prevalence, species susceptibility and implications for conservation and health strategies.

**Methods:**

We conducted a systematic review and meta‐analysis using PubMed, Web of Science and Scopus, covering studies from 2001 to 2024. Random‐effects models were employed to analyse prevalence rates, transmission pathways and regional variations, accounting for high inter‐study heterogeneity.

**Results:**

The analysis revealed substantial variation in *Brucella* detection rates, ranging from 0.25% to 100%, with an average pooled prevalence of approximately 30%. Species‐specific susceptibility was identified, with cetaceans showing the highest infection rates (52%), followed by other marine mammals (30%) and pinnipeds (18%). High prevalence rates were observed in species such as striped dolphins and beluga whales, particularly in the Mediterranean and Arctic regions. Geographic patterns indicated Asia as the region with the highest prevalence (36%), followed by Oceania and Antarctica, suggesting environmental and population density factors may influence infection rates. Smaller studies displayed a tendency toward higher reported detection rates, indicating potential publication bias.

**Conclusion:**

This meta‐analysis underscores the widespread prevalence of *Brucella* in marine mammals and the pressing need for advanced diagnostics, strengthened surveillance and targeted interventions, particularly in high‐risk regions and species. An interdisciplinary, collaborative approach including the development of molecular diagnostic tools and international research partnerships will be essential to support conservation efforts and mitigate zoonotic risks associated with marine brucellosis.

## Graphical abstract

Marine brucellosis shows high prevalence in cetaceans (52%), followed by other marine mammals (30%) and pinnipeds (18%), indicating species‐specific susceptibility. These findings highlight the need for targeted surveillance and diagnostic strategies to mitigate ecological and zoonotic risks.

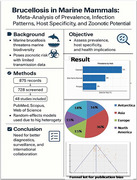

## 1 Introduction

Brucellosis is a highly infectious zoonotic disease caused by bacteria of the *Brucella* genus, affecting a wide range of both terrestrial and marine species. While traditionally associated with livestock, such as cattle, goats and sheep, the disease's impact extends into marine ecosystems, posing unique challenges for biodiversity conservation, ecological stability and public health. In ecosystems, brucellosis is primarily attributed to two species: *Brucella ceti*, which predominantly affects cetaceans, and *Brucella pinnipedialis*, which primarily infects pinnipeds (Grattarola et al. 2023). These pathogens have been detected in various marine mammals, including dolphins, whales, and seals, and have been associated with a wide spectrum of health effects ranging from asymptomatic carriage to severe neurological (e.g., meningoencephalitis), reproductive (e.g., abortions, infertility) and systemic diseases such as endocarditis and osteomyelitis (Kroese et al. 2018; Grattarola et al. 2023). As keystone species within their respective ecological niches, certain marine mammals such as dolphins and seals play a pivotal role in maintaining marine biodiversity and ecosystem balance. Their health is therefore critical, highlighting the importance of targeted research into Brucella infections affecting these populations.

The presence of marine *Brucella* species highlights the critical need for ongoing monitoring of these pathogens. Although human cases linked to marine *Brucella* infections are relatively rare, they often present with severe clinical manifestations, including neurobrucellosis and osteomyelitis, and pose significant diagnostic and therapeutic challenges (Sohn et al. 2003; McDonald et al. 2006). Individuals most at risk are those who come into close contact with infected marine mammals or their environments, such as marine biologists, veterinarians, fishermen and seafood processors (McDonald et al. 2006). Human infections from marine *Brucella* strains highlight the need for increased awareness, surveillance and diagnostic capabilities, especially in regions with frequent human–marine mammal interactions. Given the complex interplay between ecosystem and human health, the study of *Brucella* in marine mammals not only informs conservation strategies but also contributes to broader zoonotic disease prevention efforts.

Marine ecosystems are dynamic and intricate, with species interactions shaped by physical, chemical and biological factors unique to oceanic environments. Brucellosis disrupts ecosystems by impairing the health of apex and keystone species, triggering cascading effects throughout the entire food web. For instance, dolphins and whales are central to nutrient cycling and habitat maintenance, and any disease affecting these populations can alter ecosystem dynamics, potentially leading to cascading ecological effects (Kiszka et al. 2022). The high prevalence of *Brucella* in social, migratory species, such as striped dolphins and beluga whales, underscores the pathogen's adaptability to diverse ecosystems (Thompson et al. 2022). These infection patterns raise concerns about how environmental factors such as pollution, climate change and habitat degradation may influence disease susceptibility and transmission among marine species. Pollution, in particular, can weaken the immune systems of marine mammals, increasing their vulnerability to *Brucella* infections and other pathogens.

The transmission of *Brucella* in ecosystems involves complex ecological and social dynamics. Among cetaceans, which are known for their close social bonds and high levels of interaction, direct transmission is likely facilitated through activities such as mating, nursing and communal living (Vargas‐Castro et al. 2023). Similarly, pinnipeds gather in dense colonies during breeding seasons, creating ideal conditions for disease spread. Transmission from mother to offspring has been observed in both cetaceans and pinnipeds, supporting the long‐term circulation of *Brucella* within marine mammal populations (West et al. 2015). In addition, environmental contamination from bodily fluids, aborted foetuses and carcasses serves as another transmission route, with currents spreading the bacteria to new areas, potentially exposing previously uninfected populations (Korzeniewska et al. 2013). This environmental persistence highlights the challenge of controlling brucellosis in open marine settings, where pathogens can spread beyond their initial source.

Geographic variability also plays a significant role in the distribution of marine mammal brucellosis, with distinct regional patterns observed in infection rates across the globe. In European waters, particularly along the North Atlantic coast, *B. ceti* infections have been frequently reported, especially among striped dolphins and other cetacean species. This widespread prevalence underscores the significance of brucellosis in marine mammal populations, with implications for conservation and epidemiological monitoring in these regions (Garofolo et al. 2014). Social behaviours and concentrated environmental pressures help pathogens spread within and between pods. Arctic species like beluga whales and polar bears show different infection patterns due to lower population density, extreme temperatures and distinct migration. However, they remain vulnerable through infected prey, increasing health risks in ecosystems already affected by climate change and habitat loss (Dadar et al. 2022).

The adaptability of *Brucella* species to various marine hosts is demonstrated by their extensive host range, infecting cetaceans, pinnipeds and even polar bears, highlighting their ecological versatility. Studies have detected *B. ceti* in harbour porpoises, common dolphins, bottlenose dolphins and killer whales, while *B. pinnipedialis* has been observed in harbour seals, grey seals and northern fur seals (Whatmore et al. 2017). In addition, antibodies to *Brucella* have been found in polar bears and Steller's sea lions, suggesting possible infection through prey or environmental exposure (Sonne et al. 2018). This broad host range raises concerns about the ecological implications of *Brucella* infections, as it underscores the pathogen's potential to adapt to diverse marine hosts, potentially leading to cross‐species transmission and increased persistence within ecosystem.

From a conservation perspective, marine mammal brucellosis presents significant risks to vulnerable populations. *Brucella* infections can lead to reproductive issues, such as foetal loss and infertility, which are particularly concerning for species with limited reproductive rates or those already under environmental pressures. The disease's often cryptic nature complicates detection efforts, as many infected animals may show no outward signs of illness. When clinical signs do appear, they typically include reproductive failures, pneumonia and systemic infections, all of which can negatively impact population dynamics (Duncan et al. 2014). As conservation efforts increasingly focus on maintaining the health and stability of ecosystem, understanding the epidemiology of brucellosis in marine mammals becomes essential for implementing effective management and intervention strategies.

The emergence of marine mammal brucellosis raises significant public health concerns. Documented human cases linked to marine *Brucella* strains are typically associated with occupational exposure, although broader zoonotic transmission pathways may also be possible. Potential transmission routes include direct contact with infected tissues, inhalation of aerosolized particles or exposure through mucous membranes or open wounds. Given the potential severity of human brucellosis and the nonspecific nature of its symptoms, enhanced diagnostic capabilities and increased awareness are necessary, particularly in regions with high levels of human–marine mammal interaction. Human brucellosis typically presents as a febrile illness with symptoms such as fatigue, muscle and joint pain, and, in severe cases, neurological complications such as neurobrucellosis. The chronic nature of brucellosis, coupled with severe complications like endocarditis and osteomyelitis, underscores the importance of rapid and accurate diagnostics, especially in areas with marine‐related brucellosis cases (Orsini et al. 2022).

A meta‐analysis of *Brucella* infections in marine mammals reveals infection patterns, and environmental drivers, aiding conservation, public health and ecosystem management (Dadar et al. 2022). Marine mammals brucellosis presents a multifaceted challenge that intersects conservation, public health and ecological resilience. This review aims to synthesize current knowledge on *Brucella* infection patterns, host specificity and zoonotic risks in marine mammal populations. By systematically examining prevalence data, host‐specific responses and regional trends, this meta‐analysis seeks to provide actionable insights for conservation and public health stakeholders. Understanding how *Brucella* adapts to marine hosts, the environmental factors influencing its spread, and its potential for cross‐species transmission is essential for creating effective conservation strategies and mitigating zoonotic risks. This comprehensive approach aligns with One Health principles, which recognize the interconnectedness of animal, environmental and human health, and highlights the importance of interdisciplinary collaboration to address the challenges of marine mammal brucellosis.

## 2 Materials and Methods

### 2.1 Study Design and Protocol

This systematic review and meta‐analysis was conducted in accordance with PRISMA 2020 guidelines. The study protocol was developed in advance and included predefined inclusion and exclusion criteria, database search strategies and statistical approaches to ensure methodological transparency and reproducibility.

### 2.2 Literature Search Strategy

**FIGURE 1:**
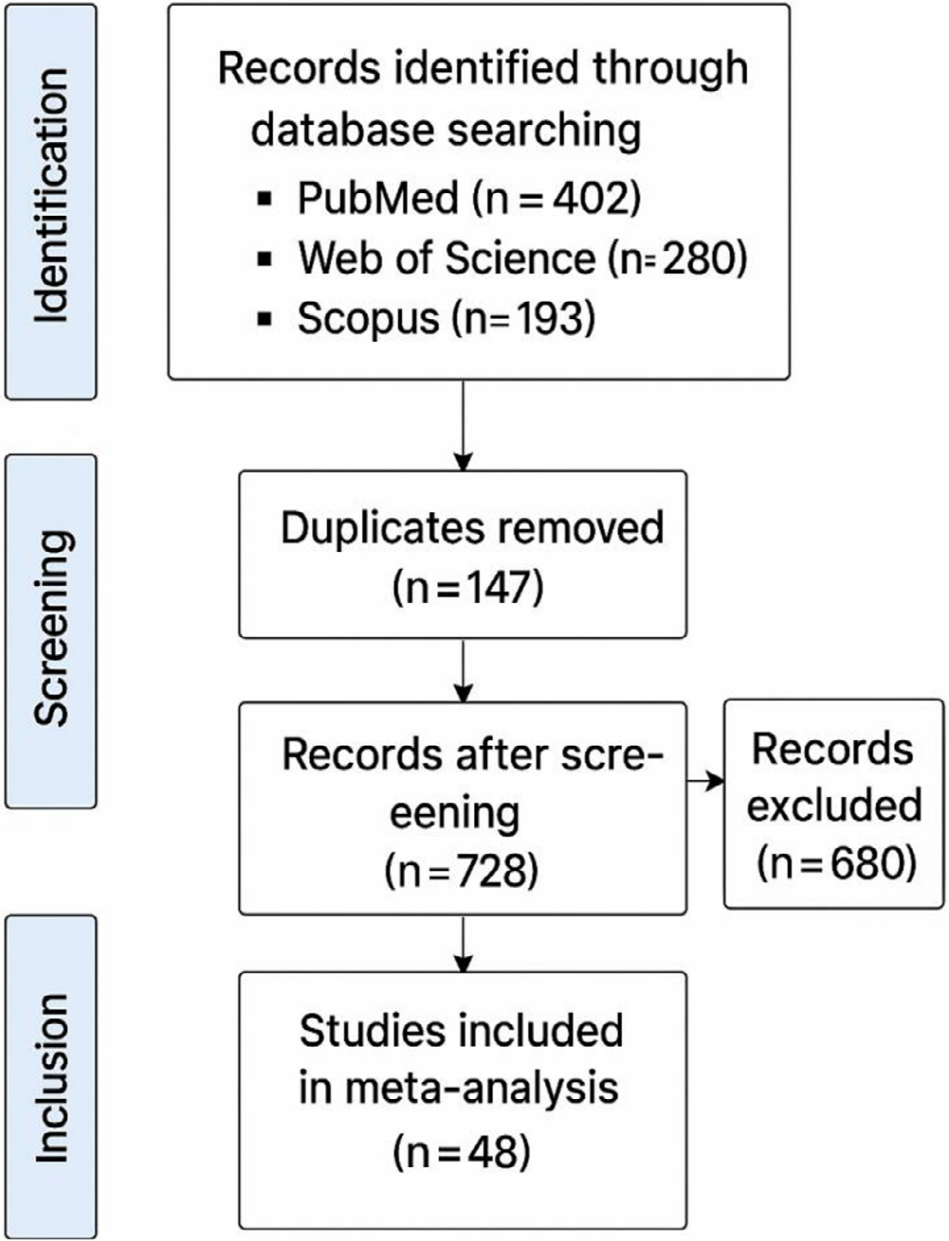
PRISMA 2020 flow diagram illustrating the study selection process for the systematic review and meta‐analysis of Brucella spp. in marine mammals. A total of 875 records were identified through database searching. After removal of 147 duplicates and screening of 728 records, 48 studies were included in the final meta‐analysis.

A comprehensive literature search was performed using three major databases: PubMed, Web of Science and Scopus, covering studies published between January 2001 and 2024. The search strategy employed Boolean operators (‘AND’, ‘OR’) to combine relevant terms related to marine mammal brucellosis. Keywords included: ‘marine brucellosis,’, ‘*Brucella* in marine mammals’, ‘*Brucella ceti*’, ‘*Brucella pinnipedialis*’ and ‘infection patterns in cetaceans and pinnipeds.’ The search retrieved a total of 875 records. After removing duplicates, titles and abstracts of the remaining records were screened for relevance. Following full‐text review and application of inclusion/exclusion criteria, 48 studies were included in the final meta‐analysis (Figure 1).

### 2.3 Inclusion Criteria

Studies were included if they met all of the following criteria:

- Published in peer‐reviewed journals.
- Reported original research with primary data.
- Focused on *Brucella* spp. infections in marine mammals (cetaceans or pinnipeds) in wild, stranded or captive contexts.
- Provided extractable data on sample size, *Brucella*‐positive cases and/or diagnostic methods.
- Written in English and with accessible full texts.

### 2.4 Exclusion Criteria

Studies were excluded if they:

- Were reviews, editorials, commentaries or conference abstracts.
- Focused exclusively on terrestrial animals or laboratory models.
- Lacked quantitative data necessary for analysis.
- Were not available in English or lacked full‐text access.

### 2.5 Study Selection Process

Two independent reviewers screened all articles. Full‐text reviews were conducted for studies meeting preliminary criteria. Disagreements were resolved by discussion or arbitration by a third reviewer. A total of 48 studies were included for meta‐analysis.

### 2.6 Data Extraction

A standardized data extraction sheet was used to record:

- Author(s) and year of publication
- Geographic location and species involved
- Sample size and diagnostic methods
- Number of *Brucella*‐positive cases
- Identified *Brucella* species (e.g., *B. ceti*, *B. pinnipedialis*)

### 2.7 Diagnostic Method Classification

The diagnostic techniques reported across studies were categorized into two main groups: direct diagnostics (including bacterial culture, PCR and immunohistochemistry [IHC]) and indirect diagnostics (including ELISA, Rose Bengal Test [RBT] and other serological assays). PCR‐based methods were the most frequently used, followed by ELISA. All included studies reported at least one of these methods with sufficient detail for data extraction. Where possible, subgroup comparisons were made based on diagnostic method to assess its influence on detection rates and pooled prevalence estimates.

### 2.8 Meta‐Analysis and Statistical Methods

Quantitative synthesis was conducted using MedCalc (v23.7.1). A random‐effects model was selected to account for expected inter‐study heterogeneity due to variation in species, geography and diagnostic methods. Pooled prevalence rates were calculated with 95% confidence intervals (CIs). Heterogeneity was assessed using the *I* ^2^ statistic:

*I* ^2^ > 75% was interpreted as substantial heterogeneity.

In our analysis, *I* ^2^ exceeded 97%, reflecting high study variability.

### 2.9 Assessment of Publication Bias

To evaluate the risk of publication bias, we used:

Funnel plots for visual inspection. Egger's regression test and Begg's rank correlation test for statistical evaluation.

Both tests indicated potential bias toward small studies with higher prevalence estimates, suggesting overrepresentation of positive findings in the published literature.

## 3 *Brucella* Species in Marine Mammals

B. ceti, a prominent marine Brucella species, primarily infects cetaceans, including dolphins, porpoises and whales (Curtiss et al. 2022). Cetacean susceptibility varies across species and regions, with infections reported globally. The bacterium is known for its widespread presence in dolphin and whale populations, which is likely influenced by their social structures and migratory behaviours. Infections are often chronic, allowing the bacteria to persist within groups and potentially spread over long distances (Whatmore et al. 2017).

Another *Brucella* species, *B. pinnipedialis*, predominantly affects pinnipeds, such as seals and sea lions, animals known for their dense, aggregative gatherings, particularly during breeding seasons (Nymo et al. 2011). Unlike cetaceans, pinnipeds alternate between land and water, affecting how infections may spread across regions. *B. pinnipedialis* primarily affects pinnipeds such as seals and sea lions and has been documented across various geographic regions, reflecting host and environmental variability (Nymo et al. 2018). Unlike *B. ceti*, which often targets neurological systems, *B. pinnipedialis* predominantly impacts reproductive and systemic health areas, including the lymph nodes and spleen. The impact of *B. pinnipedialis* on seal and sea lion mortality and population health remains under investigation. Infection prevalence varies across colonies, influenced by environmental factors, host immunity and bacterial strain diversity, with colder regions potentially experiencing distinct infection dynamics (Larsen et al. 2013).

Despite their close genetic link, *B. ceti* and *B. pinnipedialis* have unique genetic and phenotypic traits that distinguish them within the *Brucella* genus. Both share genetic similarities with terrestrial *Brucella* species like *B. abortus* and *B. melitensis* but also exhibit markers specific to marine mammal adaptation (Orsini et al. 2022). Host‐specific genetic markers suggest potential mechanisms influencing tissue tropism and chronicity, including putative immune evasion strategies; however, functional validation in marine species has not yet been achieved. *B. ceti* has a stronger tendency to invade neural tissue, possibly due to genetic adaptations that enable it to breach the blood–brain barrier in cetaceans, leading to conditions like meningitis. In contrast, *B. pinnipedialis* is adapted to target reproductive and lymphatic tissues in pinnipeds (Orsini et al. 2022). While *B*. *ceti* and *B. pinnipedialis* are currently the main *Brucella* species in marine mammals, there may be undiscovered strains in marine settings. Some *Brucella* strains found in marine mammals do not fully match the genetic profiles of *B. ceti* or *B. pinnipedialis*, suggesting ongoing evolution driven by host‐specific adaptations or environmental pressures (Vargas‐Castro et al. 2023). Emerging *Brucella* strains are supported by variations in infection patterns across different regions and hosts, indicating genetic drift or mutation in these populations. This diversity may lead to strains with modified pathogenicity or host range, with potential implications for marine mammal health and conservation. Environmental reservoirs, such as contaminated water or marine prey, might also serve as vectors for *Brucella*, allowing it to persist and spread within marine food webs (Hernández‐Mora et al. 2013). Although direct evidence of such transmission is limited, similar mechanisms have been seen in other marine pathogens, warranting further investigation into *Brucella*’s ability to persist outside mammalian hosts (Hernández‐Mora et al. 2013). Environmental factors like climate change, pollution and habitat disruption may impact *Brucella*’s evolution and spread in ecosystems. For instance, rising ocean temperatures could affect marine mammal immune function, increasing susceptibility to infections like *Brucella*. Pollutants and other stressors might further compromise marine mammal health, facilitating pathogen spread within vulnerable populations. This combination of environmental pressures and pathogen dynamics could create conditions that support the emergence of new *Brucella* strains or the expansion of existing ones within ecosystem (Labbate et al. 2016).

## 4 Infection Patterns and Pathology of Brucellosis in Marine Mammals

The spread of Brucella species among marine mammals involves a complex network of biological, environmental and ecological interactions. In addition to impacting individual health, brucellosis can alter marine mammal population dynamics and poses risks to other species, including humans, particularly those in close proximity to infected animals or sharing similar ecosystems. Understanding these interactions is essential for assessing the broader impact of Brucella on marine mammals and ecosystems, including the potential for zoonotic transmission.

### 4.1 Transmission Mechanisms of Brucellosis in Marine Mammals

Frequent close contact in cetacean social structures provides multiple opportunities for direct and indirect transmission of pathogens like Brucella. Similarly, pinniped species gather in dense colonies during breeding seasons, which naturally increases physical contact. In these close‐knit groups, Brucella can spread through both direct and indirect means. Reproductive tissues are common reservoirs for the bacteria, so mating becomes a primary pathway for transmission, particularly because the infection often targets reproductive organs. Vertical transmission from mother to offspring is a well‐documented pathway in Brucella infections among marine mammals. Infected mothers may transmit the bacteria to their young via the placenta, during birth or through nursing. This route ensures the persistence of infection within populations by facilitating early‐life exposure and enabling the pathogen to be passed through successive generations, especially in species with strong maternal care and pod structures (Vargas‐Castro et al. 2023).

Environmental factors also play a crucial role in the spread of brucellosis in marine mammals. Since *Brucella* bacteria can survive in seawater for extended periods, the pathogen is capable of spreading beyond direct contact. Marine mammals that encounter contaminated fluids or tissues from infected individuals—whether through ingestion, inhalation or contact with affected waters in affected waters—face heightened risks of infection. Areas of high population density, such as breeding or feeding grounds, become significant hotbeds for infection due to environmental persistence. Fluids, aborted foetuses and carcasses from infected animals introduce *Brucella* into the ecosystem, allowing bacteria to remain in circulation even when primary infection sources have subsided. Thus, environmental contamination serves as an ongoing reservoir for the pathogen, prolonging exposure risks for otherwise healthy individuals (Dadar et al. 2022).

Predatory species, such as polar bears and orcas, may also acquire *Brucella* infections through trophic interactions by consuming infected prey like seals and other cetaceans. This type of predator–prey transfer underscores a critical ecological pathway for *Brucella* transmission across species boundaries, extending the pathogen's influence to multiple trophic levels within ecosystem. For example, polar bears that consume *Brucella*‐infected seals may inadvertently acquire the infection, illustrating how the bacteria can cross species lines through shared habitats and food chains (Kiszka et al. 2022).

Environmental stressors, including pollutants and climate change, indirectly increase susceptibility to brucellosis in marine mammals. Pollutants such as polychlorinated biphenyls (PCBs) and heavy metals are known to weaken immune responses, making animals more vulnerable to infections like Brucella. Environmental stressors such as rising sea surface temperatures, ocean acidification and decreasing sea ice are reshaping marine ecosystems and altering prey distribution. These climate‐driven changes can force different marine species into closer contact as they shift their ranges in search of suitable habitats and food sources. For instance, warming ocean temperatures and reduced ice cover have led to latitudinal migrations and habitat overlap among cetaceans and pinnipeds, which were previously spatially or ecologically separated. In addition, ocean acidification can impact prey abundance and nutritional quality, further driving altered foraging behaviour and distribution in marine mammals (Storrie et al. 2018; Chambault et al. 2022). These shifts increase the likelihood of interspecies contact and geographic spread of infectious agents such as Brucella, heightening the risk of brucellosis transmission in naive populations (Bressem et al. 2009).

Parasitic infections, such as those caused by lungworms and liver flukes, may contribute as co‐factors in the transmission of *Brucella*. These parasites can damage host tissues, especially in cetaceans and pinnipeds, creating openings for bacterial infections. For instance, inflamed and damaged respiratory tissues due to parasitic infestations may enable *Brucella* to colonize more readily, exacerbating the infection and weakening the host's immune response. This interplay between parasitic and bacterial infections not only allows *Brucella* to propagate effectively but also increases the long‐term health impacts on affected populations, thereby sustaining the bacteria's presence within ecosystem (Rhyan et al. 2018).

### 4.2 Infection Routes and Mechanisms

*Brucella* transmission in marine mammals occurs through a combination of reproductive, respiratory and environmental pathways, contributing to its persistence and reservoir formation within marine ecosystems. Among these, reproductive transmission is particularly significant due to the pathogen's tropism for reproductive tissues. Infections such as placentitis and foetal septicaemia can lead to spontaneous abortion, infertility or neonatal death, thereby reducing reproductive success and contributing to population‐level impacts.

Respiratory transmission is considered a potential route, especially in socially cohesive species such as dolphins and seals. These animals frequently engage in close‐contact behaviours, including synchronized surfacing and vocal interactions, which may facilitate exposure to exhaled droplets containing *Brucella* spp. However, existing evidence suggests that the nasal mucosa, rather than deeper respiratory structures like the trachea or lungs, is the primary site of pathogen entry (Paiva et al. 2023). Detection of *Brucella* in lung tissue likely represents secondary colonization or haematogenous spread, rather than a primary infection route. Therefore, the role of the respiratory tract in transmission must be interpreted with appropriate caution.

The marine environment itself may also facilitate indirect transmission. Seawater can act as a medium for pathogen dispersal, with currents potentially transporting bacteria‐laden droplets or organic material between individuals. This environmental transmission is particularly relevant in regions where marine mammals aggregate in high densities, such as breeding or feeding grounds.

In addition, parasitic co‐infections may contribute to *Brucella* pathogenesis. Lungworms and liver flukes can damage host tissues, creating microenvironments that facilitate bacterial invasion and persistence (Rhyan et al. 2018). These interactions may support the establishment of long‐term infection reservoirs, particularly in the respiratory and hepatic systems.

Collectively, these transmission pathways underscore the complexity of *Brucella* epidemiology in marine mammals and the need for integrative studies that account for behaviour, environment and host–pathogen interactions.

### 4.3 Host‐Specific Pathology

The pathological impacts of Brucella infections vary significantly among host species, with B. ceti mainly affecting cetaceans and B. pinnipedialis primarily found in pinnipeds. In cetaceans, B. ceti infections primarily affect the neurological and reproductive systems, often resulting in severe clinical outcomes. Neurological involvement can include meningoencephalitis, which may present as disorientation, abnormal behaviour and, in some cases, mortality (Curtiss et al. 2022). Documented cases, such as a long‐finned pilot whale exhibiting pronounced neurological symptoms prior to stranding, demonstrate the pathogen's capacity to impair central nervous system function and disrupt social cohesion within pods (Davison et al. 2015). In addition, reproductive infections caused by B. ceti can lead to foetal loss, infertility and abortion, posing a considerable threat to population viability, especially in endangered cetacean species. In pinnipeds, B. pinnipedialis infections predominantly affect the placenta and liver, leading to reproductive and metabolic dysfunction. The development of placentitis due to infection can result in adverse pregnancy outcomes, including premature births, stillbirths or the birth of weakened offspring, all of which threaten population health by reducing survival rates of young pinnipeds. Chronic liver infections caused by Brucella can result in hepatomegaly and inflammation, impairing vital metabolic functions such as detoxification and nutrient processing both essential for marine mammal vitality and growth (Vargas‐Castro et al. 2023). Pulmonary involvement, including pneumonia, has been reported in pinnipeds infected with B. pinnipedialis, suggesting that the respiratory system may be among the affected organ systems in advanced cases. However, these findings should not be assumed to confirm primary transmission via the lungs or trachea. It is more plausible that pulmonary lesions result from haematogenous spread or secondary infection, rather than direct inhalation of the pathogen into the lower respiratory tract. This highlights the importance of differentiating between primary entry routes and secondary organ involvement when interpreting Brucella‐related pathology in marine mammals.

*Brucella* infections in marine mammals are propagated through intricate transmission routes, including direct contact, environmental exposure and trophic interactions. The pathogen's ability to exploit reproductive, respiratory and environmental niches within ecosystem highlights its adaptability and resilience. Pathological effects, particularly on reproductive and neurological health, underscore *Brucella*’s substantial impact on population dynamics and ecosystem stability.

### 4.4 Subclinical Infections

Subclinical infections, where infected individuals exhibit no overt symptoms, are a significant aspect of *Brucella* epidemiology in marine mammals. Such infections are often asymptomatic but represent a continuous reservoir of the pathogen, complicating efforts to track and manage its spread within populations. Asymptomatic *Brucella* infections are common in marine populations, with infected individuals carrying and potentially shedding bacteria without visible symptoms (Buckle et al. 2017). This phenomenon complicates disease surveillance, as standard observational methods may miss asymptomatic carriers, requiring more advanced diagnostic techniques, such as serological and molecular testing, to detect and quantify infection prevalence accurately. The high incidence of subclinical infections suggests that the pathogen's impact on marine mammal populations may be underestimated, as asymptomatic infections can still reduce fitness over time, affecting reproductive success, immunity and overall health (Kroese et al. 2018). Asymptomatic carriers play a critical role in *Brucella* persistence within ecosystem, shedding bacteria into the water and contaminating shared environments, particularly in high‐density areas like breeding and feeding grounds. These carriers facilitate pathogen adaptability by maintaining a stable reservoir, allowing *Brucella* to evolve and potentially develop more virulent strains over time (Sánchez‐Sarmiento et al. 2019). As a result, addressing both symptomatic and asymptomatic carriers is essential for effective disease management in marine mammals.

## 5 Key Findings

### 5.1 Variability in *Brucella* Detection in Marine Mammals

Table 1 reveals diverse detection rates of *Brucella* spp. across marine mammals and related species, shaped significantly by the diagnostic method, sample type and host species.

**TABLE 1:** Prevalence and detection methods of Brucella spp. in marine mammals and related species across geographic regions.

Species	Sample type	Country/region	Diagnostic method	Total sample size	No. of positive	Detection rate (%)	References
Marine mammals	Blood	North America	Serology	2470	94	3.8	Nielsen et al. (2001)
Marine mammals	Tissues	France	PCR	33	33	100.0	Cloeckaert et al. (2001)
Marine mammals	Serum	Spain	Serology	76	35	46.05	Van Bressem et al. (2001)
Polar bears	Plasma	Svalbard and Barents Sea	Slow Agglutination of Wright (SAW), EDTA modified SAW, Rose Bengal, protein A ELISA	297	16	5.4	Tryland et al. (2001)
Striped dolphins	Brain tissue and others	Scottish Coast, United Kingdom	Immunohistochemistry and serology	3	3	100.0	González et al. (2002)
Minke whales	Serum	North Pacific	Serology, PCR	83	19	22.89	Ohishi et al. (2003)
Seal	Serum and tissues	nan	Serology and culture	74	7	9.46	Maratea et al. (2003)
Minke whales (*Balaenoptera acutorostrata*)	Testes	Western North Pacific	PCR, histopathology, immunohistochemistry	22	10	4.5	Ohishi et al. (2004)
Hooded seals	Various tissues	North Atlantic Ocean	Culture, serology	29	11	37.9	Tryland et al. (2005)
Pacific bottlenose dolphin (*Tursiops aduncus*)	Serum	Solomon Islands	TAT, ELISA, immunoblotting	58	40	69.0	Tachibana et al. (2006)
Hawaiian monk seal	Serum	Northwestern Hawaiian Islands	Various serology tests	332	83	25.0	Aguirre et al. (2007)
Striped dolphin (*Stenella coeruleoalba*)	Cerebrospinal Fluid	Costa Rica	Immunofluorescence, ELISA, PCR	10	7	70.0	Hernández‐Mora et al. (2008)
Harbour porpoises	Blood	Bay of Fundy, Canada	cELISA, FPA	170	2	1.2	Neimanis et al. (2008)
Seal (*Halichoerus grypus*) and other marine mammals	Various organs	German North Sea	Serology	768	50	6.51	Prenger‐Berninghoff et al. (2008)
Striped dolphin (*Stenella coeruleoalba*)	Brain, various tissues	Costa Rica	PCR, immunofluorescence, serology	17	16	94.1	Hernández‐Mora et al. (2009)
Striped dolphin (*Stenella coeruleoalba*)	Serum	Mediterranean region	Serology	14	14	100.0	González‐Barrientos et al. (2010)
Antarctic fur seal (*Arctocephalus gazella*)	Serum	Antarctic	ELISA, Rose Bengal	102	31	30.4	Jensen et al. (2013)
Harbour seal (*Phoca vitulina*)	Serum	Washington, USA	Serology	1314	100	7.6	Lambourn et al. (2013)
Bottlenose dolphin (*Tursiops truncatus*)	Lung, brain	South Carolina, USA	Real‐time PCR	178	55	30.9	Wu et al. (2014)
Northern fur seal (*Callorhinus ursinus*)	Placenta and serum	St. Paul Island, Alaska	qPCR	400	1	0.25	Duncan et al. (2014)
California sea lion (*Zalophus californianus*)	Blood, vaginal Mucus, and milk	Gulf of California, Mexico	Rose Bengal, PCR	22	5	22.7	Ávalos‐Téllez et al. (2014)
Striped dolphin (*Stenella coeruleoalba*)	Brain and other tissues	Western Mediterranean	Culture, PCR, histopathology	3	3	100.0	Isidoro‐Ayza et al. (2014)
Harbour porpoises (*Phocoena phocoena*)	Tissue samples	Dutch Coast	PCR	112	7	6.3	Maio et al. (2014)
Harbour seals	Tissues	Central California	PCR	58	2	3.4	Greig et al. (2014)
Northern fur seal (*Callorhinus ursinus*)	Placenta and serum	Pribilof Islands, Alaska	PCR, serology	159	7	4.4	Duncan et al. (2015)
Fur seal	serum	PUNTA SAN JUAN, PERU	Serology	28	15	53.7	Jankowski et al. (2015)
Bottlenose dolphins (*Tursiops truncatus*)	Lung tissues	Virginia and others USA	PCR	81	18	22.0	Wu et al. (2016)
Hector's dolphin (*Cephalorhynchus hectori*)	Various tissues	New Zealand	Histology, PCR	27	7	26.0	Buckle et al. (2017)
Northern fur seal (*Callorhinus ursinus*)	Placenta and serum	Hokkaido, Japan	ELISA, Western blot	307	88	28.7	Abe et al. (2017)
Asian sea otter (*Enhydra lutris lutris*)	Rectal swabs	Bering Island, Russia	PCR	78	3	3.8	Burgess et al. (2017)
Grey seal (*Halichoerus grypus*)	Liver	Baltic Sea	PCR, Culture	122	3	2.5	Hirvelä‐Koski et al. (2017)
Bearded seal (*Erignathus barbatus*)	Blood and lymphnode	Chukchi Sea, Svalbard, Bering Sea, Bering Strait, and Scotland	Serology and multilocus sequence typing (MLST)	200	22	11.0	Foster et al. (2018)
Beluga whale (*Delphinapterus leucas*)	Serum	Russian Arctic	ELISA	4	3	75.0	Ohishi et al. (2018)
Ringed seal (*Pusa hispida*)	Blood serum	Baltic Sea	Rose Bengal, ELISA	21	2	9.5	Sonne et al. (2018)
Atlantic cod (*Gadus morhua*)	Atlantic cod tissue	Pacific Northwest, specifically Washington State, United States.	qPCR	18	9	50.0	Norman et al. (2018)
Seals	Alaskan waters, United States	Serum	Serology	1643	345	21.0	Nymo et al. (2018)
Clymene dolphin (*Stenella clymene*)	Brain tissue	Northeast Brazil	PCR	13	1	7.7	Attademo et al. (2018)
Striped dolphins (*Stenella coeruleoalba*)	Italian coastline and Canary Islands (Spain)	Central Nervous System (CNS) tissue samples	Serology	9	7	77.8	Di Francesco et al. (2019)
Various marine mammals	Tissues, blood	Brazilian Coast	Serology and PCR	129	14	10.9	Sánchez‐Sarmiento et al. (2019)
Striped dolphin (*Stenella coeruleoalba*)	Brain tissue	Italian Seas	ELISA, PCR, culture	8	6	75.0	Garofolo et al. (2020)
Melon‐headed whale (*Peponocephala electra*) and other cetacean species	Serum	Coast of Japan	Serology	32	1	3.1	Ohishi et al. (2020)
Bottlenose dolphins (*Tursiops truncatus*)	Tissue samples	South Carolina, USA	qPCR	282	90	32.0	Wayne et al. (2020)
Antarctic fur seal (*Arctocephalus gazella*)	Serum	Antarctic Territory	RBT, CFT, ELISA	17	6	35.3	Retamal et al. (2021)
Svalbard white whale (*Delphinapterus leucas*)	Serum samples	Svalbard region, Arctic	Serology	27	16	59.0	Nymo et al. (2022)
Spotted seal (*Phoca largha*)	Serum	Hokkaido, Japan	ELISA	118	32	27.1	Ohishi et al. (2022)
Beluga whale	Blood, tissue	Alaska, USA	Serology (ELISA), rtPCR, bacterial culture	167	102	61.08	Thompson et al. (2022)
Striped dolphin	Brain, various tissues	Italy	Microbial isolation, PCR, histopathology	24	24	100.0	Grattarola et al. (2023)
Australian fur seal	Aborted foetuses, placentas	Australia	PCR, histopathology, immunohistochemistry	171	9	5.3	Gardner et al. (2024)

*Note*: Direct methods include PCR and bacterial culture; indirect methods include serological tests such as ELISA and RBT.

The diagnostic techniques employed across studies were broadly categorized into two groups:

Direct diagnostics, including polymerase chain reaction (PCR), bacterial culture and IHC

Indirect diagnostics, such as ELISA, RBT and serum agglutination tests (SAT)

Among these, PCR was the most commonly used method (in ∼60% of studies), often in combination with tissue sampling. Studies using PCR consistently reported higher detection rates, ranging from 25% to 100%, particularly in tissue‐based samples. For example, striped dolphins off the Scottish coast showed 100% positivity in brain tissue, and marine mammals in France demonstrated 100% tissue positivity using PCR.

In contrast, serological methods, primarily used in blood or serum samples, generally produced lower detection rates. Harbour porpoises in Canada had the lowest recorded detection at 1.2% using ELISA. These lower rates likely reflect the limitations of serology in detecting subclinical or early‐stage infections, and the inability to distinguish between past exposure and current infection.

Culture‐based methods, though highly specific, were less frequently applied due to the need for high‐quality fresh samples and extended incubation times. Their detection rates ranged from 5% to 35%, depending on sample quality, host species and laboratory conditions.

Sample type also played a crucial role in influencing detection rates. Across all diagnostic categories, tissue samples especially brain, lung and lymphatic tissues yielded higher positivity than blood or serum. This suggests a preference of *Brucella* spp. for specific tissue tropism and highlights the limitations of relying solely on blood‐based diagnostics in marine wildlife studies.

Species‐specific variation was also observed. Striped dolphins consistently showed higher prevalence (up to 100%) than other marine species such as harbour seals (6.51%), underscoring possible differences in host susceptibility, immune response or behavioural ecology.

Diagnostic method, sample type and host species collectively influence *Brucella* detection rates. PCR in tissue samples appears to offer the most sensitive approach for identifying active infections, while serology underestimates prevalence, particularly in asymptomatic or early‐stage cases.

### 5.2 Diagnostic Precision and Methodological Heterogeneity

**FIGURE 2:**
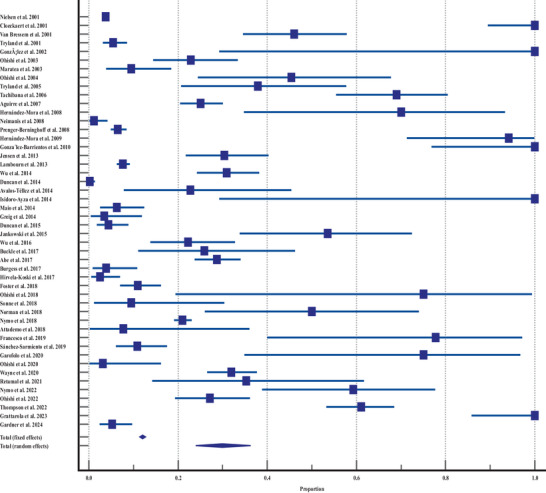
Forest plot of brucellosis detection rates by study, showing proportion and confidence intervals across various studies.

The forest plot (Figure 2) underscores variability across studies, with detection proportions spanning from 0.25% (Duncan et al. 2014) to 100% (multiple studies including Grattarola et al. 2023). Fixed‐effects models estimate the proportion of *Brucella* detection in marine mammals at approximately 12%, while random‐effects models suggest nearly 30%, reflecting high inter‐study variability (Table 2). Studies with smaller sample sizes, such as Ohishi et al. (2018), show broader CIs, signalling lower precision, whereas larger studies like Nielsen et al. (2001) offer narrower intervals. The *Q*‐test (*Q* = 1869.86, *p* < 0.0001) and an *I* ^2^ value of 97.49% further confirm substantial heterogeneity among studies (Table 3).

**TABLE 2:** Detection rates of brucellosis in marine mammals across studies: sample size, proportion, confidence intervals and study weights.

Study	Sample size	Proportion (%)	95% CI	Weight (%)	
				Fixed	Random
Nielsen et al. (2001)	2470	3.806	3.086–4.637	23.81	2.39
Cloeckaert et al. (2001)	33	100.000	89.424–100.000	0.33	2.09
Van Bressem et al. (2001)	76	46.053	34.548–57.875	0.74	2.25
Tryland et al. (2001)	297	5.387	3.110–8.601	2.87	2.36
González et al. (2002)	3	100.000	29.240–100.000	0.039	1.06
Ohishi et al. (2003)	83	22.892	14.380–33.417	0.81	2.26
Maratea et al. (2003)	74	9.459	3.888–18.524	0.72	2.25
Ohishi et al. (2004)	22	45.455	24.386–67.790	0.22	1.97
Tryland et al. (2005)	29	37.931	20.687–57.740	0.29	2.05
Tachibana et al. (2006)	58	68.966	55.456–80.461	0.57	2.21
Aguirre et al. (2007)	332	25.000	20.433–30.019	3.21	2.36
Hernández‐Mora et al. (2008)	10	70.000	34.755–93.326	0.11	1.64
Neimanis et al. (2008)	170	1.176	0.143–4.185	1.65	2.33
Prenger‐Berninghoff et al. (2008)	768	6.510	4.870–8.493	7.41	2.38
Hernández‐Mora et al. (2009)	17	94.118	71.311–99.851	0.17	1.87
González‐Barrientos et al. (2010)	14	100.000	76.836–100.000	0.14	1.79
Jensen et al. (2013)	102	30.392	21.672–40.287	0.99	2.29
Lambourn et al. (2013)	1314	7.610	6.235–9.179	12.67	2.39
Wu et al. (2014)	178	30.899	24.201–38.246	1.72	2.33
Duncan et al. (2014)	400	0.250	0.00633–1.385	3.86	2.37
Ávalos‐Téllez et al. (2014)	22	22.727	7.821–45.370	0.22	1.97
Isidoro‐Ayza et al. (2014)	3	100.000	29.240–100.000	0.039	1.06
Maio et al. (2014)	112	6.250	2.550–12.454	1.09	2.30
Greig et bal. (2014)	58	3.448	0.420–11.908	0.57	2.21
Duncan et al. (2015)	159	4.403	1.788–8.860	1.54	2.32
Jankowski et al. (2015)	28	53.571	33.870–72.489	0.28	2.04
Wu et al. (2016)	81	22.222	13.734–32.828	0.79	2.26
Buckle et al. (2017)	27	25.926	11.114–46.285	0.27	2.03
Abe et al. (2017)	307	28.664	23.671–34.075	2.97	2.36
Burgess et al. (2017)	78	3.846	0.800–10.831	0.76	2.25
Hirvelä‐Koski et al. (2017)	122	2.459	0.510–7.018	1.19	2.30
Foster et al. (2018)	200	11.000	7.023–16.180	1.94	2.34
Ohishi et al. (2018)	4	75.000	19.412–99.369	0.048	1.19
Sonne et al. (2018)	21	9.524	1.175–30.377	0.21	1.95
Norman et al. (2018)	18	50.000	26.019–73.981	0.18	1.89
Nymo et al. (2018)	1643	20.998	19.052–23.049	15.84	2.39
Attademo et al. (2018)	13	7.692	0.195–36.030	0.13	1.76
Di Francesco et al. (2019)	9	77.778	39.991–97.186	0.096	1.59
Sánchez‐Sarmiento et al. (2019)	129	10.853	6.062–17.538	1.25	2.31
Garofolo et al. (2020)	8	75.000	34.914–96.815	0.087	1.54
Ohishi et al. (2020)	32	3.125	0.0791–16.217	0.32	2.08
Wayne et al. (2020)	282	31.915	26.513–37.702	2.73	2.36
Retamal et al. (2021)	17	35.294	14.210–61.672	0.17	1.87
Nymo et al. (2022)	27	59.259	38.798–77.610	0.27	2.03
Ohishi et al. (2022)	118	27.119	19.346–36.075	1.15	2.30
Thompson et al. (2022)	167	61.078	53.240–68.515	1.62	2.33
Grattarola et al. (2023)	24	100.000	85.753–100.000	0.24	1.99
Gardner et al. (2024)	171	5.263	2.435–9.756	1.66	2.33
Total (fixed effects)	10,330	12.049	11.428–12.691	100.00	100.00
Total (random effects)	10,330	29.933	23.954–36.275	100.00	100.00

**TABLE 3:** Test for heterogeneity.

*Q*	1869.8580
DF	47
Significance level	*p* < 0.0001
*I* ^2^ (inconsistency)	97.49%
95% CI for *I* ^2^	97.11–97.82

### 5.3 Regional and Host‐Specific Variability in *Brucella* Detection

Geographic variation plays a major role in the prevalence of Brucella among marine mammals. For example, Antarctic fur seals demonstrated a relatively high positivity rate of 30.4% using ELISA, while northern fur seals in Alaska showed a much lower prevalence of 4.4%. Similarly, spotted seals in Japan exhibited a 27.1% detection rate, indicating notable regional differences. Bottlenose dolphins sampled in the USA had a prevalence of 30.9% in lung tissue, whereas grey seals from the Baltic Sea showed a markedly lower rate of 2.5%. These findings highlight how pathogen prevalence may be shaped by regional ecological conditions, host density and environmental stressors (Table 1).

### 5.4 Temporal Trends in *Brucella* Detection

**FIGURE 3:**
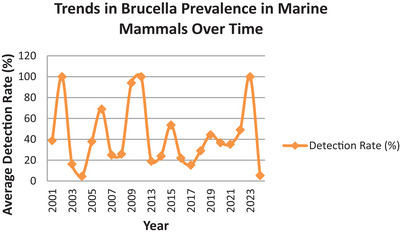
Trends in *Brucella* prevalence in marine mammals over time.

The analysis of detection rates over time reveals a cyclical pattern in *Brucella* prevalence among marine mammals, with significant peaks reaching up to 100% in certain years, followed by marked declines (Figure 3). These fluctuations could suggest episodic outbreaks, possibly driven by environmental changes, variations in *Brucella* exposure, or shifts in sampling and diagnostic techniques. Temporal trends in detection may also correlate with broader ecological changes, such as climate‐related stressors, migratory shifts or alterations in marine mammal populations. Understanding these trends is crucial for identifying potential triggers and forecasting future outbreaks, emphasizing the importance of monitoring environmental factors that could influence prevalence over time.

### 5.5 Host‐Specific Detection Rates

**FIGURE 4:**
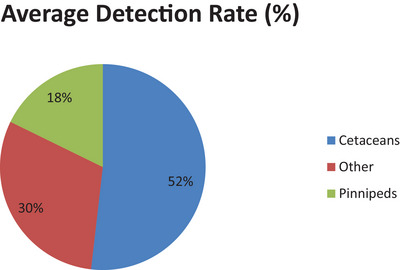
Average detection rate in cetaceans versus pinnepeds.

The analysis reveals distinct host‐specific patterns in *Brucella* detection rates. Cetaceans exhibit the highest average prevalence at 52%, followed by other marine mammal groups at 30%, and pinnipeds at 18% (Figure 4). This distribution suggests that cetaceans may serve as key reservoirs of infection, likely due to their frequent social interactions, such as group foraging, pod cohesion and extended maternal care, which facilitate pathogen transmission. Their wide‐ranging migratory behaviour may further increase exposure to diverse environmental reservoirs of *Brucella*.

In contrast, pinnipeds lower average detection rate may reflect different transmission dynamics. These species typically gather in high densities only during specific periods like the breeding season, limiting prolonged contact that supports widespread transmission. Such ecological and behavioural differences underscore the importance of tailoring disease surveillance and intervention strategies to the life history and social structure of each host group.

A notable case supporting this host‐specific pattern involves a short‐beaked common dolphin (*Delphinus delphis*) in the UK, which presented with *B. ceti* associated meningoencephalitis and arthritis (Davison et al. 2013). This case illustrates the severity of infection in cetaceans and reinforces their epidemiological significance in marine brucellosis dynamics (Davison et al. 2013).

### 5.6 Geographic Variability in *Brucella* Prevalence

**FIGURE 5:**
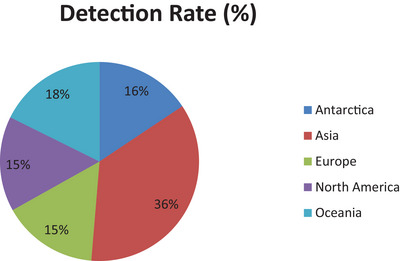
Detection rate (%) of brucellosis in marine mammals across different continents.

The geographic distribution of *Brucella* prevalence shows significant variability across marine regions. According to compiled data, Asia reported the highest detection rate at 36%, followed by Oceania (18%), Antarctica (16%) and both Europe and North America (15%) (Figure 5). These differences likely reflect a combination of ecological and environmental factors, as well as variations in sampling effort, diagnostic techniques and study intensity across regions, which may influence detection rates and prevalence estimates.

Regions such as Asia and Oceania may support higher transmission due to the greater density and diversity of susceptible marine mammal species, along with unique environmental pressures like pollution or coastal development. In contrast, areas reporting lower prevalence may reflect genuine differences in exposure risk or simply variation in surveillance coverage, sample size or diagnostic sensitivity (Figure 5).

This variability highlights the importance of implementing region‐specific monitoring and control measures. Tailored strategies that account for local ecosystem dynamics, species composition and anthropogenic influences are essential for effective management of *Brucella* infections in marine mammal populations worldwide.

### 5.7 Publication Bias Assessment in *Brucella* Detection Studies

**FIGURE 6:**
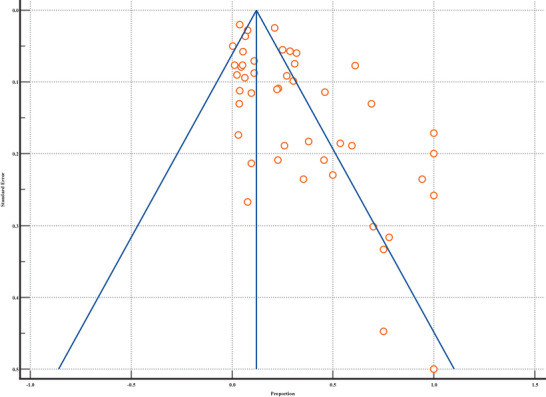
Funnel plot of brucellosis detection proportions in marine mammals, illustrating publication bias based on standard error and proportion distribution.

The results of the heterogeneity test (*Q* = 1869.8580, DF = 47, *p* < 0.0001) indicate significant variability across the included studies, with an *I* ^2^ value of 97.49% (95% CI = 97.11–97.82), suggesting that most of the observed differences in *Brucella* detection rates are due to factors other than sampling error. This substantial heterogeneity underscores the need for caution when interpreting the pooled estimates. Publication bias, as assessed through Egger's test (intercept = 4.9136, *p* = 0.0001), reveals a strong indication that smaller studies with higher detection proportions may be overrepresented in the literature, likely due to the higher likelihood of publication for statistically significant findings (Table 4). In addition, Begg's test (Kendall's Tau = 0.2213, *p* = 0.0265) provides weaker evidence of bias, although it still suggests some level of asymmetry. This is further supported by the funnel plot, which shows a clear bias toward studies reporting higher detection rates, particularly on the right side of the plot (Figure 6). This asymmetry, along with the increased variability in smaller studies, highlights the potential overestimation of *Brucella* prevalence in marine mammals and the importance of considering publication bias in meta‐analytical interpretations.

**TABLE 4:** Publication bias.

Egger's test	
Intercept	4.9136
95% CI	2.5485–7.2788
Significance level	*p* = 0.0001
Begg's test	
Kendall's Tau	0.2213
Significance level	*p* = 0.0265

## 6 Host Specificity and Reservoirs of Brucellosis in Marine Mammals

Brucella infection patterns in marine mammals differ substantially between cetaceans and pinnipeds, driven by variation in behaviour, physiology and ecological interactions. Research across multiple regions has highlighted differences in transmission dynamics and host susceptibility, with certain species playing a more prominent role in maintaining Brucella within marine ecosystems.

Cetaceans, such as striped dolphins (Stenella coeruleoalba) and bottlenose dolphins (Tursiops truncatus), consistently demonstrate high infection rates, underscoring their potential role as primary reservoirs of B. ceti. Their social structure, long‐distance migration and pod‐based living likely facilitate widespread transmission across and within populations. Pinnipeds, by contrast, are primarily infected by B. pinnipedialis, which targets reproductive and lymphoid tissues. Although typically showing lower prevalence rates, pinnipeds form dense aggregations during the breeding season, creating episodic opportunities for efficient transmission.

Beluga whales (Delphinapterus leucas), particularly those in Arctic regions, have also been identified as important hosts, often harbouring the pathogen without overt clinical signs (Table 5). Their ecological role as potential long‐term carriers supports the idea of species‐specific contributions to Brucella maintenance in different marine environments. A study in Northeast Brazil emphasized how ecological and behavioural variation across marine mammal species may influence transmission dynamics, reinforcing the need for regionally tailored assessments (Sousa et al. 2021).

**TABLE 5:** Comparative summary of infection patterns by host species adapted from multiple studies on *Brucella* infection patterns in marine mammals, including (Nielsen et al. 2001; Cloeckaert et al. 2001; Van Bressem et al. 2001; Tryland et al. 2001), and others as cited in Table 1.

Host species	Primary *Brucella* species	Key infected tissues	Clinical manifestations	Infection rate
Striped dolphin (*Stenella coeruleoalba*)	*Brucella ceti*	Brain, reproductive tissues	Meningoencephalitis, abortion	Up to 100% in certain regions
Bottlenose dolphin (*Tursiops truncatus*)	*Brucella ceti*	Brain, reproductive organs	Neurological symptoms, reproductive failure	30%–70%
Harbour seal (*Phoca vitulina*)	*Brucella pinnipedialis*	Reproductive organs, lymph nodes	Infertility, reproductive complications	5%–15%
Northern fur seal (*Callorhinus ursinus*)	*Brucella pinnipedialis*	Placenta, liver, lymphoid tissues	Reproductive issues, hepatomegaly	4%–10%
Beluga whale (*Delphinapterus leucas*)	*Brucella ceti*	Blood, lymphoid tissues	Variable, asymptomatic carriers common	Up to 75% in Arctic regions

### 6.1 Host‐Specific Patterns in Environmental Interactions

Cetaceans and pinnipeds exhibit distinct ecological behaviours that influence the persistence and spread of Brucella infections. In cetaceans, strong social bonds and extended maternal care facilitate sustained transmission within pods through close and repeated contact. In contrast, pinnipeds typically congregate at densely populated breeding sites, where temporary but intense interactions create seasonal transmission hotspots. In addition, in some species such as Alaska's beluga whales, exposure through contaminated prey or environmental sources suggests trophic and habitat‐driven transmission pathways that differ from the more socially mediated spread observed in cetaceans (Thompson et al. 2022).

### 6.2 Adaptation to Marine Hosts

The adaptation of Brucella spp., particularly B. ceti and B. pinnipedialis, to marine mammal hosts reflects a high degree of bacterial specialization shaped by ecological and physiological pressures. Marine mammals possess distinct traits such as elevated dissolved oxygen in the bloodstream and increased myoglobin for deep diving that require pathogens to modify their survival strategies. It is hypothesized, based on experimental findings and genetic inference, that *Brucella* strains adapt cellular functions such as respiration and membrane integrity to withstand oxygen‐rich and high‐pressure marine environments. However, direct evidence in marine mammal remains limited (Vargas‐Castro et al. 2023).

Immune adaptation also plays a central role. Marine mammals exhibit streamlined immune systems suited to their specific habitats, often with reduced exposure to diverse pathogens (Sousa et al. 2021). Based on genomic studies and model‐based inference, marine *Brucella* species may evade host immune responses through potential modifications in outer membrane proteins, though direct confirmation in marine mammal hosts is still limited.

The host specificity of B. ceti and B. pinnipedialis likely evolved through ecological separation and genetic divergence. With limited contact between marine and terrestrial hosts, B. ceti became specialized for cetaceans and B. pinnipedialis for pinnipeds. This specialization is supported by genetic markers unique to marine Brucella strains, indicating evolutionary changes tailored to marine environments (Orsini et al. 2022).

Pathological patterns further illustrate these adaptations. In cetaceans, B. ceti predominantly infects reproductive tissues, contributing to abortions and reduced fertility. In pinnipeds, B. pinnipedialis more frequently targets the respiratory system, aligning with adaptations to their diving physiology and fluctuating oxygen levels (Curtiss et al. 2022). These distinct infection routes and host preferences highlight the evolutionary pressures driving Brucella specialization in marine ecosystems.

### 6.3 Potential for Cross‐Species Transmission

Although B. ceti and B. pinnipedialis are primarily adapted to marine mammals, occasional spillovers into terrestrial species suggest a degree of zoonotic potential (Tryland et al. 2001). For example, polar bears have exhibited serological evidence of Brucella exposure, likely through the consumption of infected seals (Rode et al. 2024). This trophic interaction illustrates how marine pathogens can cross into terrestrial predators, potentially affecting their health, reproductive success or hunting efficiency.

Experimental infection studies have demonstrated that marine Brucella strains are capable of infecting terrestrial livestock species such as cattle and sheep, albeit with generally lower virulence compared to terrestrial strains (Perrett et al. 2004). However, these findings are based on controlled laboratory conditions and may not accurately reflect natural transmission dynamics or pathogenicity in field settings. As such, they should be considered as indicative of potential host range rather than conclusive evidence of cross‐species transmission in ecological contexts. These findings emphasize the importance of monitoring potential spillover pathways, particularly in areas where marine mammal byproducts are incorporated into animal feed or where human–animal interfaces are frequent.

While cross‐species transmission events appear rare, their ecological and health implications underscore the need for integrated disease management strategies, especially in Arctic and coastal regions where ecosystem boundaries are increasingly blurred.

### 6.4 Reservoir Role of Marine Mammals in Brucella Maintenance and Spread

Certain marine mammal species serve as reservoirs for Brucella, playing a key role in maintaining and disseminating the pathogen within and across populations. Cetaceans, particularly dolphins and some whale species, are primary reservoirs for B. ceti, while seals and sea lions maintain B. pinnipedialis populations (Vargas‐Castro et al. 2023). Their social structures and migratory behaviours support sustained transmission. Frequent group interactions among dolphins, including mating and coordinated movement, create ideal conditions for B. ceti spread. Long‐distance migrations by dolphins and whales can transport the bacteria across regions, contributing to genetic diversity and expanding its ecological reach.

Pinnipeds also act as important reservoirs. During breeding seasons, their dense colonies facilitate both vertical transmission—from mothers to pups—and horizontal spread among adults. This is reflected in cases of B. pinnipedialis in two bottlenose dolphins along Spain's Cantabrian coast, where evidence supports maternal transmission and regional environmental persistence (Vargas‐Castro et al. 2023). Infections in pinnipeds commonly target the respiratory and reproductive systems, aligning with their colony‐based transmission dynamics.

Beyond intra‐species transmission, the reservoir role of marine mammals has broader ecological implications. Infected individuals may influence pathogen flow across trophic levels (Sharma and Sumithra 2023). For instance, apex predators such as polar bears consuming infected prey could acquire Brucella, facilitating interspecies transmission. Environmental shifts driven by climate change such as ocean warming and habitat loss may also alter migration patterns and aggregation sites, creating new transmission routes and expanding the pathogen's geographic range (Dadar et al. 2022).

Understanding the reservoir dynamics of marine mammals is critical for developing effective surveillance and control strategies, especially as environmental changes intensify the complexity of marine brucellosis management.

## 7 Zoonotic Potential and Health Implications of Marine Mammal Brucellosis

Brucellosis, once associated primarily with terrestrial animals, is increasingly recognized in marine ecosystems, where it presents a zoonotic risk. B. ceti and B. pinnipedialis, the two species identified in marine mammals, have been linked to human cases involving systemic infections like neurobrucellosis, spondylitis and osteomyelitis (Sohn et al. 2003; Sierra et al. 2019; Mackie et al. 2020). The *ST27* genotype is particularly concerning due to its virulence and immune evasion capabilities (Cvetnić et al. 2016; Orsini et al. 2022).

Most human infections result from occupational exposure veterinarians, marine mammal handlers, researchers, seafood processors and laboratory workers are at heightened risk due to direct contact, aerosol inhalation or mucosal exposure (Hunt et al. 2008; Isidoro‐Ayza et al. 2014; Zygmunt et al. 2010). In areas with high seafood consumption, ingestion of raw or undercooked seafood presents a secondary transmission route (Hernández‐Mora et al. 2013).

Though marine *Brucella* generally do not persist in open water, localized contamination and shoreline exposure cannot be ruled out (Cvetnić et al. 2016). Given the range of symptoms and severity, especially with ST27 infections, enhanced diagnostic capacity and awareness are essential for early detection and treatment. Human cases often present with nonspecific symptoms, making differential diagnosis challenging without molecular confirmation.

Effective public health response requires a One Health approach, combining biosafety, marine mammal health monitoring and international collaboration due to migratory species crossing national boundaries. Risk communication, occupational safety protocols and continued surveillance remain vital, particularly in coastal communities.

## 8 Diagnostic Challenges and Advances in *Brucella* Detection in Marine Mammals

The detection and diagnosis of Brucella infections in marine mammals present distinct challenges due to the biology of marine Brucella species, unique host characteristics, and environmental constraints. Unlike terrestrial species such as B. abortus, B. melitensis and B. suis, marine strains like B. ceti and B. pinnipedialis exhibit different biochemical profiles and growth requirements, complicating their isolation and identification through conventional laboratory methods. For example, a harbour porpoise (Phocoena phocoena) infected with B. ceti exhibited skin ulcers, pneumonia and necrotic tissue, but diagnosis required IHC and bacteriology to confirm infection—underscoring the diagnostic difficulty when clinical signs are subtle or non‐specific (Sakyi et al. 2023).

Traditional diagnostics—including bacterial culture and serological tests—are limited in sensitivity and specificity. Culture‐based methods are often hampered by the slow growth rate and specialized environmental needs of marine Brucella (Islam et al. 2019). In addition, contamination and suboptimal sample quality from stranded or bycaught animals reduce diagnostic success (Vargas‐Castro et al. 2023; Sakyi et al. 2023).

Serological testing, widely used in terrestrial hosts, faces notable limitations in marine mammals. Cross‐reactivity with Yersinia and Francisella may yield false positives, while species‐specific serological assays are lacking (Sánchez‐Sarmiento et al. 2019). Marine Brucella antibodies may not respond reliably in standard assays, and such tests cannot distinguish between active infections and past exposures. This limits their utility for monitoring current infection status or estimating prevalence accurately (Orsini et al. 2022).

To address these issues, molecular techniques have become central to marine Brucella detection. A notable case from Japan confirmed B. ceti sequence type 27 in a bottlenose dolphin (Tursiops truncatus) with osteomyelitis—an identification made possible through multilocus sequence typing (MLST) and PCR, showing genetic similarity with strains from minke whales in the same region (Ueno et al. 2020). These advanced diagnostics are crucial for identifying strains in geographically isolated populations and assessing epidemiological links.

PCR and its variations—real‐time PCR, qPCR and multiplex PCR—allow for species‐level identification by targeting specific Brucella genes, often in degraded samples or those with low bacterial loads (Sidor et al. 2013). Multiplex PCR can differentiate B. ceti from B. pinnipedialis (Mayer‐Scholl et al. 2010), while PCR‐RFLP and multilocus variable number tandem repeat analysis (MLVA) enhance resolution and track transmission pathways (Islam et al. 2019). MLVA offers insights into strain variation and potential movement across regions, and SNP typing further enables high‐resolution analysis of genetic differences, revealing host adaptation, transmission dynamics and zoonotic potential.

These molecular methods have transformed diagnostics by improving detection speed, specificity and the ability to trace infection sources within ecosystems. The ability to differentiate strains and identify species‐specific markers is essential for understanding transmission routes and guiding conservation and public health strategies (Maquart et al. 2009).

Whole‐genome sequencing (WGS) represents a major breakthrough, providing complete genetic profiles that reveal virulence traits, antimicrobial resistance and host specificity. WGS allows researchers to trace Brucella movement between hosts and across geographic areas, aiding in the identification of infection reservoirs and potential cross‐species transmission routes (Shallom et al. 2012).

Complementing WGS, sensor‐based technologies are emerging as powerful diagnostic tools. Electrochemical and optical biosensors enable real‐time detection of Brucella DNA or antigens in field settings. These tools require minimal sample preparation and are suited for remote locations where laboratory facilities are limited (Pei et al. 2019; McCutcheon et al. 2019). Their portability and sensitivity allow for rapid, in situ health assessments of stranded or at‐risk marine mammals.

The integration of WGS with machine learning and the application of biosensors offer promising advances in marine Brucella diagnostics. Together, these tools support early detection, real‐time monitoring and proactive health management in marine mammal populations.

### 8.1 Conservation Implications of Marine Brucellosis

Brucellosis poses a significant threat to endangered and vulnerable marine mammal populations, where even minor disease outbreaks can have severe impacts due to small population sizes and limited reproductive capacity. Infections caused by B. ceti and B. pinnipedialis may lead to reproductive failure, neurological disorders and increased mortality. These outcomes are particularly detrimental for species with restricted ranges or already facing anthropogenic pressures. For example, the critically endangered Maui's dolphin, with fewer than 100 individuals remaining in New Zealand's coastal waters, is at high risk; an outbreak of brucellosis could further reduce reproductive rates and overall resilience (Hamner et al. 2014). Chronic infections or post‐infection immune suppression can leave surviving individuals vulnerable to other diseases and environmental stressors, exacerbating population decline. In areas with frequent human interaction, higher brucellosis prevalence also increases the risk of zoonotic transmission, adding complexity to conservation strategies (McFee et al. 2020).

Environmental factors such as pollution, climate change and habitat degradation further intensify disease susceptibility. Pollutants—including heavy metals, plastics and endocrine disruptors—can impair immune function and reproductive health, creating conditions favourable for pathogen persistence. These stressors compromise marine mammals' ability to resist infections and may enhance disease severity within affected populations. Climate change adds further pressure, altering prey availability, migration patterns and sea temperatures, which may influence Brucella distribution and virulence. Warming waters can facilitate pathogen proliferation, while shifting habitats may introduce Brucella into new populations and geographic areas (Desforges et al. 2016; Dadar et al. 2022).

The potential for co‐infection with other pathogens adds another dimension to the conservation challenge (Raga et al. 1997). Immunocompromized marine mammals may harbour multiple infectious agents, with synergistic effects that accelerate disease progression and mortality. In a study by Groch et al. (2020), dolphins infected with both Brucella spp. and morbillivirus exhibited worsened clinical outcomes, illustrating how co‐infections complicate diagnosis, treatment and recovery. Such interactions underscore the need for a broader health management approach that accounts for overlapping disease risks.

Comprehensive conservation strategies must integrate disease surveillance, environmental monitoring and targeted health assessments. Limiting human–animal interactions, protecting critical habitats and addressing pollution and climate threats are crucial to reducing stressors that facilitate brucellosis outbreaks. Conservation policies should also incorporate pathogen screening in rescue and rehabilitation programs to prevent disease reintroduction into wild populations. A holistic, interdisciplinary approach is vital for enhancing resilience and promoting recovery in threatened marine mammal species.

## 9 Future Prospects in Brucellosis Surveillance and Detection

To address brucellosis in marine mammals and its zoonotic potential, future efforts must emphasize enhanced surveillance, diagnostic innovation and targeted public health strategies. Expanding surveillance is critical for early detection, especially in regions with high human–marine mammal interactions and populations facing significant environmental stressors. A global, coordinated surveillance network that monitors both marine mammal health and brucellosis prevalence could provide comprehensive insights into infection trends, transmission pathways and emerging risks. This network should leverage molecular tools and automated data‐sharing platforms to ensure timely updates for conservation and public health stakeholders. Advances in diagnostics, particularly through molecular and genomic tools like PCR, MLVA and WGS, are essential for improving detection accuracy. These technologies enable precise identification of *Brucella* strains, facilitating early intervention and the potential for disease tracking across regions and host species. Furthermore, real‐time, portable diagnostics, such as sensor‐based technologies, could revolutionize field testing in remote marine environments, supporting proactive disease management and allowing for rapid response to outbreaks. For public health, enhanced zoonotic surveillance in at‐risk coastal communities and occupational groups is paramount. Education initiatives that raise awareness of brucellosis risks in the seafood industry, tourism and research sectors should emphasize best practices in animal handling, food preparation and preventive hygiene measures. In addition, interdisciplinary research on vaccine development for high‐risk marine species holds promise for mitigating brucellosis impact on vulnerable populations. By integrating these efforts, we can support marine conservation, protect at‐risk human populations and strengthen ecosystem resilience in the face of growing environmental pressures. These future‐focused strategies underscore the importance of a One Health approach, recognizing the interconnectedness of human, animal and environmental health in managing the complexities of marine brucellosis.

## 10 Conclusion

This meta‐analysis provides a comprehensive examination of the extensive impact and complex epidemiology of brucellosis in marine mammals, primarily associated with *Brucella* species. Our review reveals notable gaps in existing data, particularly concerning prevalence across various species, regional patterns and the intricate dynamics of transmission within diverse ecosystems. By systematically analysing infection patterns, we identified high prevalence rates in certain species, such as striped dolphins and beluga whales, and in regions like Asia and the Arctic, where environmental stressors such as climate change and pollution may further influence disease susceptibility and spread. Addressing these data gaps will require a multifaceted strategy that includes expanded surveillance, particularly in under‐studied regions, alongside advanced diagnostic methods tailored to detect marine *Brucella* strains more accurately. Specific measures such as developing species‐appropriate vaccines and strengthening international research collaborations are crucial for managing the impact of brucellosis on vulnerable marine populations and mitigating zoonotic risks, especially among high‐exposure groups like fishermen, veterinarians and marine researchers. This study underscores the interconnectedness of ecosystem health, animal health and human health, an alignment with One Health principles that recognizes the need for interdisciplinary efforts to manage zoonotic diseases. Collaborative international research efforts, supported by advanced technologies like WGS and biosensor‐based diagnostics, will be essential to advancing conservation strategies and public health interventions. These measures, guided by the insights of this meta‐analysis, can play a vital role in safeguarding both marine biodiversity and human communities that rely on oceanic ecosystems, highlighting the importance of proactive, holistic approaches to disease management in marine environments.

## Author Contributions

**Nasrin Sultana Tonu**: conceptualization, investigation, writing – review and editing. **Md. Anowar Hossain**: conceptualization, methodology, writing – original draft, writing – review and editing. **Md. Sadequl Islam**: conceptualization, methodology, data curation, investigation, supervision, writing – original draft, writing – review and editing. **Bristi Kona Debnath**: data curation, methodology, writing – review and editing.

## Conflicts of Interest

The authors declare no conflicts of interest.

## Peer Review

The peer review history for this article is available at https://www.webofscience.com/api/gateway/wos/peer‐review/10.1002/vms3.70557.

## Data Availability

Data will be provided upon request from the corresponding author.
